# Effectiveness of magnetite nanoparticles for the removal of DNA of multidrug-resistant* Escherichia coli* from municipal wastewater

**DOI:** 10.1007/s11356-024-35098-5

**Published:** 2024-09-30

**Authors:** Mike O. Ojemaye, Omobola O. Okoh, Anthony I. Okoh

**Affiliations:** 1 SAMRC Microbial Water Quality Monitoring Center, University of Fort Hare, Eastern Cape, Alice, 5700 South Africa; 2 Applied Environmental and Microbiology Research Group, Department of Biochemistry and Microbiology, University of Fort Hare, Eastern Cape, Alice, 5700 South Africa; 3 Department of Pure and Applied Chemistry, University of Fort Hare, Eastern Cape, Alice, 5700 South Africa

**Keywords:** Magnetite, Nanoparticles, *Escherichia coli*, Adsorption, DNA, Antibiotic resistance, Aqueous solution

## Abstract

Water is an important component of life and plays a vital role in human and animal lives, and because of these essential roles of water to life, access to quality water and in adequate quantity becomes imperative. Subsequently, water/wastewater systems have become established as reservoirs of antimicrobial resistance determinants in the environment. In this study, we carried out the synthesis and characterization of magnetite nanoparticles for use in the removal of genomic DNA of antibiotic resistant *Escherichia coli* isolate in aqueous system. The synthesis of this nanoparticle was achieved by using precipitation method and characterization of the material was conducted by using Fourier transform infrared spectroscopy (FTIR), scanning electron spectroscopy (SEM), electron diffraction spectroscopy (EDS), and vibrating sample magnetometry (VSM). The SEM analysis revealed that this material is spherical in shape, while the FTIR spectra revealed Fe–O vibrational band at ~ 450 cm^−1^ confirming the success of the synthesis of this material. The magnetite nanomaterial showed efficiency for the removal of the bacterial DNA from water with a maximum removal capacity of 45.5 ng g^−1^. The effect of pH (*q*_*e*_ max = 55 ng g^−1^ @ pH = 10), time (*q*_*t*_ max @ 180 min), DNA concentration (DNA concentration of 185 ng/mL, *q*_*e*_ max = 45.5 ng g^−1^), and adsorbent weight (% adsorption max = 61.65% @ 0.025 g) suggest that adsorption conditions influence the removal of DNA by this material. In addition, kinetic study shows that the removal of bacterial DNA obeyed pseudo 2nd order indicating that adsorption process was achieved by bimolecular interaction between magnetite and antibiotics resistant bacterial DNA. We conclude that magnetite nanoparticle appears to be a potential candidate for the removal of nucleic acids and antimicrobial resistance determinants in water/wastewater treatment.

**Supplementary Information:**

The online version contains supplementary material available at 10.1007/s11356-024-35098-5.

## Introduction

Antibiotic resistance is a pressing global health crisis, driven in part by the widespread distribution of antibiotic resistance genes (ARGs) in various environmental matrices, including water and wastewater (Shao et al. 2018). The presence of ARGs in these environments poses a serious threat to public health, as they can potentially transfer to human pathogens, rendering antibiotics ineffective in treating infections (Zainab et al. 2020). As the antibiotic resistance crisis deepens, it becomes imperative to develop innovative strategies to mitigate the dissemination of ARGs in water and wastewater (Ojemaye et al. 2020).

The proliferation of antibiotic resistance is a multifaceted problem, with numerous pathways through which ARGs can disseminate into the environment. A critical aspect of this problem is the horizontal transfer of ARGs, where bacteria exchange genetic material, including ARGs, via mechanisms such as conjugation, transduction, and transformation. Environmental waters and wastewater serve as reservoirs for these pathogenic and commensal bacteria harboring ARGs. As a result, the presence of bacterial DNA harboring ARGs in these matrices has been widely documented. For example, studies by Pallares-Vega et al. (2019), Anthony et al. (2018), Adefisoye and Okoh (2016), and Mao et al. (2015) reported various ARGs in wastewater treatment plants, surface waters, and even drinking water sources, highlighting the potential public health threat associated with waterborne ARGs.

Efforts to mitigate this challenge have primarily focused on traditional treatment methods, such as chemical disinfection (Owoseni et al. 2017) and filtration (Slipko et al. 2019; Spit et al. 2022). However, these methods are often inadequate in effectively removing ARGs, and in some cases, they may even induce selective pressure leading to the emergence of more antibiotic-resistant strains (Varga et al. 2012). Therefore, the search for innovative approaches to address this challenge is imperative.

While a substantial body of research has explored the presence of ARGs in environmental matrices, their removal has received relatively less attention. One promising approach is the use of nanoparticles for the removal of DNA harboring antibiotic resistance genes, which represents an emerging field of research with the potential to offer sustainable and efficient solutions (Aruguete et al. 2013; Ma et al. 2016).

Magnetite nanoparticles, characterized by their magnetic properties and high surface area, offer a novel and promising approach to address this issue. These nanoparticles have the potential to selectively bind to DNA fragments, including those containing ARGs, and subsequently facilitate their removal from water. The utilization of magnetite nanoparticles for the removal of DNA harboring antibiotic resistance genes presents a multifaceted rationale. Firstly, these nanoparticles exhibit superparamagnetic behavior, which means that they become magnetized in the presence of an external magnetic field and return to their non-magnetic state when the field is removed (Ojemaye et al. 2017a, b; Ojemaye et al. 2018). This property allows for easy separation and recovery of the nanoparticles after adsorption, ensuring minimal secondary pollution and cost-effective reusability. Furthermore, magnetite nanoparticles have a high specific surface area, providing ample binding sites for DNA molecules. This characteristic enhances their capacity to adsorb DNA fragments, including those carrying ARGs. The nanoparticles’ large surface area-to-volume ratio also enables efficient mass transfer, enhancing the kinetics of DNA adsorption and, by extension, the overall removal process (Xu et al. 2012). Magnetite nanoparticles are inherently stable and biocompatible, reducing concerns about potential toxicity or environmental impact (McCarthy and Weissleder 2008). The biocompatibility of these nanoparticles is essential when considering their application in water and wastewater treatment (Xu et al. 2012), as they should not introduce harmful substances or negatively impact the aquatic ecosystem.

Many studies have explored the use of nanoparticles to remove bacterial DNA conveying ARGs from water and wastewater. For instance, a published paper demonstrated the successful adsorption of plasmid DNA harboring extended-spectrum beta-lactamase (ESBL) genes using iron oxide nanoparticles for the detection of arsenic (Liu and Liu 2014). These nanoparticles were able to adsorb the plasmid DNA effectively, for arsenic detection. Similarly, Ezeuko et al. (2022a) published a paper on the removal of DNA harboring ARGs from hospital wastewater using mesoporous silica etched nanoparticles, reducing the potential for horizontal gene transfer and providing evidence of the feasibility of this approach to real-world scenarios. In addition, Anthony et al. (2022) reported the use of CeO nanoparticles for the removal of DNA harboring ARGs from tap water. Despite this, to the best of our knowledge, no study has been conducted on the removal of bacterial DNA harboring ARGs from water using magnetite nanoparticles. The novelty of this study involves utilizing magnetite nanoparticles (NPs) to efficiently remove bacterial DNA harboring ARGs from water offers a promising solution with significant potential benefits including the separation of magnetite material from water after treatment by magnetic means and removal of DNA by magnetite by electrostatic means. This study aims to explore the capabilities and limitations of magnetite NPs for removal of bacterial DNA harboring ARGs, to determine the mechanisms by which magnetite nanoparticles adsorb and remove DNA from water systems. The novelty of this study also includes the influence of adsorption parameters on the removal of DNA harboring ARGs from water.

In summary, the increasing prevalence of antibiotic resistance genes in water and wastewater is a growing public health concern. However, this research field is still evolving and requires further investigation to address practical and environmental considerations. The findings from this study will contribute to a deeper understanding of the potential of magnetite nanoparticles as a promising material for combating antibiotic resistance determinants in water and wastewater contributing to the development of innovative strategies for eliminating antibiotic resistance in the environment.

## Experimental

### Materials

Iron(II)Chloride hexa hydrate (FeCl_2_.6H_2_O), Iron(III)Chloride tetra hydrate (FeCl_3_.4H_2_O), and sodium hydroxide (NaOH) were supplied by Merck Chemicals, South Africa. Nuclease-free water was supplied by Thermo Fischer. All materials supplied were of analytical grade and used as received.

### Synthesis of magnetite nanoparticles

Solutions of FeCl_2_.6H_2_O and FeCl_3_.4H_2_O in molar ratio 1:2 were prepared and reacted together under nitrogen for 1 h. Sodium hydroxide solution (8 M) was added slowly to the reaction until the pH of the reacting mixture was 11 and the temperature of the reaction was raised to 75 °C. The reaction was kept stirring for 4 h under nitrogen atmosphere. The precipitate obtained was collected by centrifugation, washed thrice and dried for 12 h at 105 °C. The dried sample was calcined at 450 °C for 3 h (Maaz et al. 2007).

### Characterization of magnetite nanoparticles

The textural and adsorption capabilities of magnetite were assessed using different techniques. Fourier transform infrared spectrophotometer (FTIR) was employed for the confirmation of the vibrational bands of the material by placing a small sample on FT-IR spectrometer (Perkin Elmer Universal ATR sampling accessory spectrum 100). The morphology of the material was obtained by using scanning electron microscope (SEM) (JOEL JSM-6390); this was done by placing a small sample of the material on a carbon double sided tape on a stub and coated with Au/Pd for a clearer image. Magnetic measurements of the material were taken with a vibrating samples magnetometry (VSM) system of 14 T at temperature values of 1.8 to 310 K. Thermal analysis of magnetite was performed under nitrogen at 20 mL min^−1^ using Perkin Elmer TGA 4000 analyzer. The determination of point of zero charge (pHpzc) was evaluated as reported in one of our published works Ojemaye et al. (2017a, b) and by Oyetade et al. (2016). This is important to determine the electrical charge density on the adsorbent material.

### Extraction of DNA of multidrug-resistant Escherichia coli isolate

In this study, multidrug-resistant *Escherichia coli* isolated from municipal wastewater in our previous study (Anthony et al. 2020) was used. The *Escherichia coli* isolate was resistant against eight antibiotic drugs including erythromycin, ciprofloxacin, tetracycline, ampicillin, rifampicin, chloramphenicol, cephalothin, and imipenem (Anthony et al. 2020). DNA extraction was achieved using boiling method (Dashti et al., 2009) and stored at – 20 °C. The concentration and purity of the extracted DNA were determined using NanoDrop One AZY2019951, firmware version 145. For confirmation of resistance genes, polymerase chain reaction (PCR) assay was conducted using the primers reported by (Anthony et al. 2020) and showed that the *E*. *coli* isolate harboured two resistance genes (gryB and udiA). The PCR products were observed in agarose gel (2% w/v) stained with ethidium bromide and thereafter imaged by using Alliance BioDoc-It system.

### Adsorption experiments

Adsorption experiments were done using orbital shaker operating at 150 rpm. Then, 25 mL bottles were employed, containing 10 mL of a solution with varied DNA concentrations (185 ng/mL, 165 ng/mL, and 145 ng/mL) derived from the stock solution (257 ng/mL). These DNA working concentrations were adopted because of the concentration of DNA found in municipal wastewater plants from published data (Owoseni and Okoh 2017). The solution included as-synthesized samples of magnetite nanoparticles and 200 mM NaCl salt solution. The presence of NaCl is to ensure adsorption of DNA by magnetite since both DNA and magnetite are negatively charged. To validate the obtained experimental data, the experiments were conducted in duplicate.

The effect of pH was investigated by shaking 0.02 g of magnetite samples into a 10 mL working solution containing 165 ng/mL DNA concentration at room temperature. The initial pH of the working solution was adjusted to values between 2 and 10 using dropwise additions of dilute solutions of NaOH or HCl. After 360 min of contact time, the DNA concentration in the supernatant was determined to assess the effects of pH on DNA carrying ARGs adsorption onto magnetite nanoparticles.

The DNA adsorbed per unit mass (*qe*) of magnetite was calculated according to Eqs. (1) and (2) from Fu et al. (2015):

1 $${q}_{e}=V\frac{\left({C}_{o}- {C}_{i}\right) }{m}$$

2 $$\text{Removal efficiency}\left(\%\right)=\frac{\left(C_o-C_i\right)}{C_o}\times100$$

where *qe* is the adsorption capacity (ng/g), *V* is the volume of adsorbate solution (mL), *m* is equal to adsorbent mass (g), and *C*_*o*_ and *C*_*i*_ are the initial and final concentrations of DNA in ng/mL. For effect of time, the equation of *qt* is the same as *qe*.

For effects of contact time (5–360 min) and DNA carrying ARGs concentrations (185 ng/mL, 165 ng/mL, and 145 ng/mL), experiments were conducted by keeping other conditions (pH and adsorbent dose) constant. Kinetic experiments were conducted at pH 6.00 without adjustment, using DNA concentrations of 185 ng/mL, 165 ng/mL, and 145 ng/mL. The experiments covered different time intervals from 5 to 360 min. The remaining DNA concentrations in the solution were determined using a dsDNA NanoDrop One AZY2019951, firmware version 145 by placing the supernatant in a cuvette and read at 260 nm. The data obtained were fitted into three kinetic models including pseudo 1st order, pseudo 2nd order, and Elovich (SI Table 1). Additionally, the impact of adsorbent amount (0.005, 0.01, 0.015, 0.02, and 0.025 g) was determined to ascertain DNA removal capacity by the adsorbent. The effect of cations (Na^+^, Ca^2+^, and Al^3+^) competing with DNA for adsorption was investigated to understand the affinity of magnetite to DNA in solutions containing varying cations.

### Analysis of data

Data analysis and plotting were done using *originPro* Graphing and Analysis version 8.5 as well as Microsoft Excel 2021.

## Results and discussion

### Characterization of magnetite nanoparticles

To ascertain the complete success of the synthesis of magnetite nanoparticles, this session reports some characterization techniques prior to the use of this material for the adsorption of bacterial DNA carrying ARGs from aqueous solution.

#### FTIR analysis

**Fig. 1:**
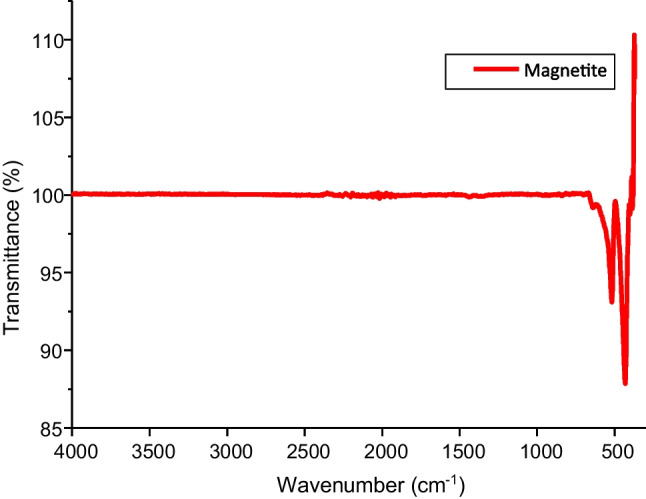
FTIR spectra of magnetite nanoparticles

FTIR analysis indicated that magnetite nanoparticles were synthesized in this study (Fig. 1). Vibrational bands around 480 cm^−1^ assigned to metal–oxygen bond in the form of Fe–O for magnetite was observed in this spectrum. This spectrum shows that this material is pure and free of impurities as no other vibrational peaks were observed except the one found around 500 cm^−1^. This observation is similar to the one observed by (Malekpour and Khodadadi 2016).

#### SEM and EDX analysis

**Fig. 2:**
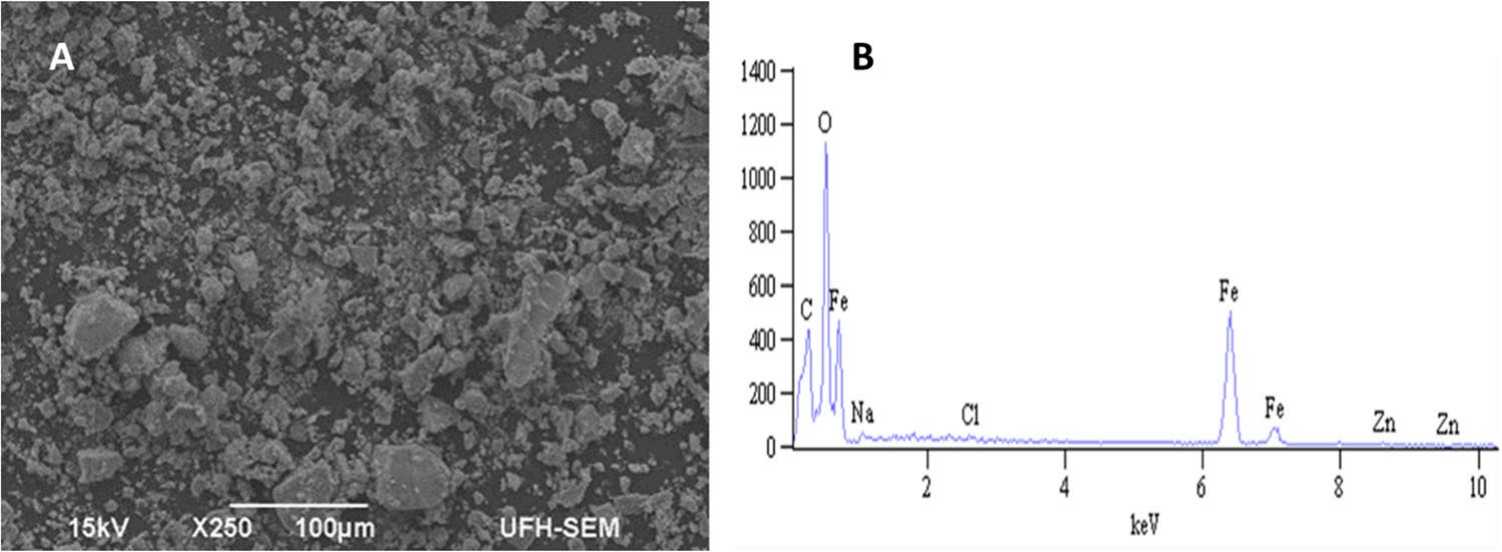
Images of **A** SEM and **B** EDX of magnetite

SEM analysis conducted at 100-µm magnification for the synthesized nanomaterial shows that an irregular spherical shaped material was obtained (Fig. 2A). The result indicates that even though slight agglomeration was observed in the micrograph of magnetite (Fig. 2A), the shape and formation were found to be uniform. This observation compliments the results previously published for magnetite nanoparticles (Wang et al. 2015). EDX analysis shown in Fig. 2B indicates that magnetite synthesized in this study is mainly composed of Fe atoms (78%) with intense signals. This result confirmed that this material is a magnetite, and it is pure.

#### TGA and VSM analysis

**Fig. 3:**
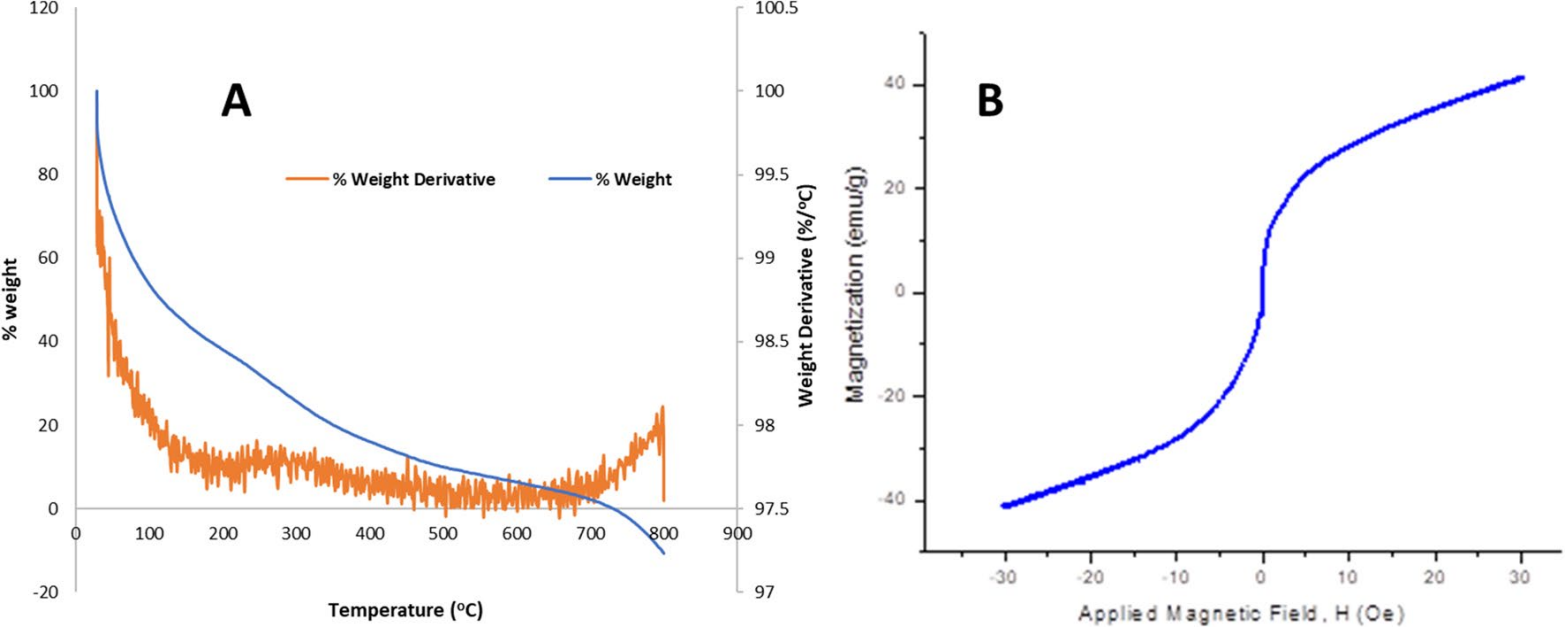
Spectra showing **A** thermogravimetric analysis and **B** vibrating sample magnetometry analysis of magnetite

Thermal analysis of the synthesized material is presented in Fig. 3A. This analysis is important to determine the stability and purity of the synthesized magnetite. The thermograph shows that less than 3% weight loss was obtained when this material was subjected to about 800 °C heat. About 1% weight loss was observed between 0 and 200 °C; this is usually ascribed to decomposition of the material due to loss of water. This 1% weight loss is insignificant and compliments the FTIR spectrum (Fig. 1) which shows no broad vibrational peaks around 2900–3000 cm^−1^ for the presence of water molecules in this material. The weight derivative analysis showed that this material became stable from temperature range 300–800 °C with no major decomposition observed (Fig. 3A). The weight derivative analysis showed weight loss from temperature range 30–300 °C which compliments the result observed in the TGA curve.

Vibrating sample magnetometry analysis (VSM) of this material shows that this material is highly magnetic with an emu/g of 40 when the applied magnetic field increases to 30 Oe and vice versa (Fig. 3B). This magnetization measurement indicates that this material at room temperature showed no hysteresis which is a signal of its superparamagnetic property. The implication of this result is that the separation of magnetite from aqueous solution can be achieved by magnetic means.

### Batch adsorption studies

#### Effect of pH

**Fig. 4:**
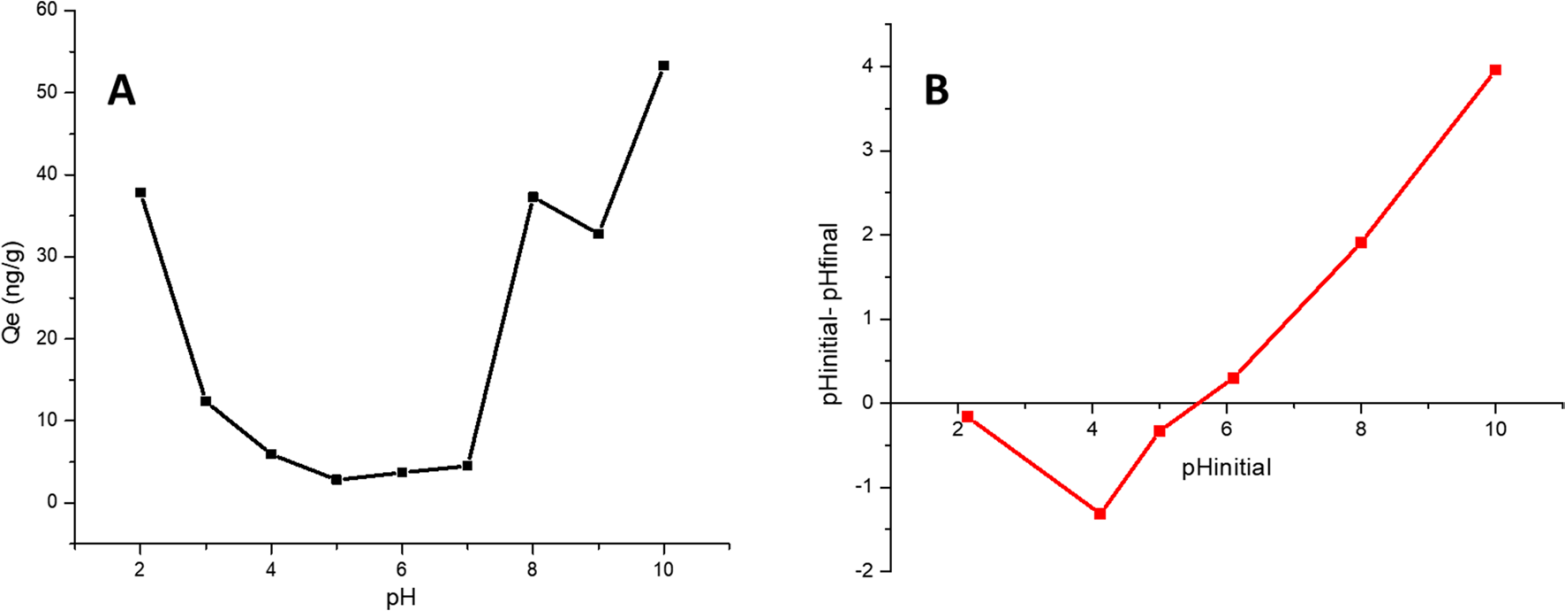
**A** Effect of pH on the removal of DNA from water **B** Point of zero charge of magnetite nanoparticles (Conditions: DNA Conc: 165 ng/mL, 20 °C, 200 mM NaCl, 0.02 g magnetite dosage and 6 h contact time)

The effect of pH on the removal of bacterial DNA harboring ARGs from water was evaluated between pH 2 and 10 using 10 mL volume of 165 ng/mL initial DNA concentration, 200 mM NaCl, 0.02 g magnetite dosage for 6 h at room temperature (Fig. 4A). The result shows that adsorption capacity (*q*_*e*_) was at maximum (*q*_*e*_ = 37.86 ng/g) at pH 2 and began to decrease (*q*_*e*_ = 4.52 ng/g) as the pH of the DNA solution increased to 7. Interestingly, at pH 8, the adsorption capacity abruptly increased to 37.27 ng/g and thereafter increased as the pH is raised to 10. The reason for this trend is that naturally, at pH values 5 to 9, DNA bonds are very stable but at lower and higher pH values, their molecules are destabilized (Li et al. 2012). For instance, at pH values less than 5, DNA losses its purine bases, the phosphodiester bonds in DNA molecule break while at pH values greater than 9, there is an abundance of hydroxide ions caused by alkaline denaturation (Xu et al. 2019). In addition, the electrostatic interaction between the surface of magnetite and the phosphate end of DNA at lower pH could have resulted in the higher adsorption capacity of magnetite for DNA removal at pH 2. Similar observation was recently reported by Anthony et al. (2020).

In Fig. 4B, the point of zero charge for the synthesized nanomaterial is presented. The point of zero charge (PZC) is a critical concept in understanding the adsorption behavior of materials like magnetite nanoparticles, especially in relation to the removal of bacterial DNA harboring antibiotic resistance genes (ARGs) from water. It is the pH at which the surface of the magnetite nanoparticles has a neutral net charge. This means that at this specific pH, the number of positive charges equals the number of negative charges on the material’s surface. For the magnetite used in this study, the PZC is identified at pH 5.7, as shown in Fig. 4B. From the plot of initial pH against change in pH, it was observed that the magnetite material has a total surface charge of 5.7. The implication of this result is that at pH lower than 5.7, that adsorbent has a net positive charge. DNA molecules, which have a negatively charged phosphate backbone, will be attracted to the positively charged magnetite surface through electrostatic interactions. This explains why the adsorption capacity is highest at pH 2 (*q*_*e*_ = 37.86 ng/g). The strong electrostatic attraction at low pH enables more DNA molecules to adhere to the magnetite surface. The bed structure of magnetite at these lower pH values likely allows for the aggregation of DNA molecules onto the positively charged surface. The compact nature of the magnetite bed and its surface charge distribution at low pH facilitate closer contact between the DNA molecules and the magnetite, enhancing adsorption. As the pH increases toward the PZC, the net surface charge of the magnetite becomes more neutral. The electrostatic attraction between the magnetite and the negatively charged DNA diminishes, leading to a decrease in adsorption capacity. This trend is observed as the pH increases from 2 to 7, with a minimum adsorption capacity (*q*_*e*_ = 4.52 ng/g) at pH 7. At this pH, the DNA is stable, but the weaker interaction with the near-neutral magnetite surface leads to reduced adsorption. The near-neutral charge on the magnetite surface may lead to less effective packing of DNA molecules within the magnetite bed structure, further reducing adsorption. The reduction in electrostatic forces makes the surface less accommodating to the negatively charged DNA, leading to lower adsorption efficiency. At pH higher than 5.7, the net charge on the surface of the adsorbent is negative. One might expect this to repel the negatively charged DNA molecules, reducing adsorption. However, the observed trend shows an increase in adsorption capacity at pH 8 (*q*_*e*_ = 37.27 ng/g) and higher pH values. This can be explained by the destabilization of DNA at higher pH values due to the abundance of hydroxide ions, which may weaken the DNA structure and facilitate its interaction with the negatively charged magnetite surface despite the electrostatic repulsion. Additionally, other non-electrostatic forces, such as van der Waals forces or hydrogen bonding, might contribute to the adsorption process at these higher pH levels. The bed structure of magnetite at these higher pH values may accommodate the destabilized DNA molecules differently. Even though the surface is negatively charged, the altered state of DNA might enable it to interact with the magnetite surface through non-electrostatic forces. The structural changes in DNA at these pH levels might allow it to penetrate deeper into the magnetite bed, resulting in increased adsorption capacity.

The bed structure of the magnetite nanoparticles, combined with the variation in surface charge at different pH levels, plays a crucial role in DNA adsorption. At pH 2, the highly positive charge on the surface creates strong electrostatic attractions with DNA, maximizing adsorption (Kooh et al. 2017; Kua et al. 2020). As the pH approaches the PZC, the reduction in surface charge leads to a corresponding decrease in adsorption. Beyond the PZC, despite the negative charge, the weakened and destabilized DNA structure at higher pH levels still allows for significant adsorption, possibly due to other interactive forces (Fig. 4).

#### Effect of contact time

**Fig. 5:**
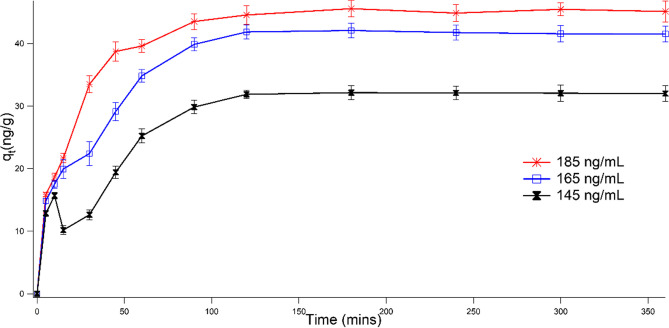
Effect of contact time on the removal of multidrug resistant bacterial DNA from aqueous solution (Conditions: DNA concentrations of 185 ng/mL, 165 ng/mL, and 145 ng/mL;20 °C; 200 mM NaCl; 0.02 g magnetite dosage and pH 6)

The effect of contact time for the removal of bacterial DNA harboring ARGs from wastewater by magnetite which was conducted at three different DNA concentrations by fixing the pH at 6 and adsorbent weight at 20 mg is presented in Fig. 5. It can be observed that adsorption of DNA is affected by time. Fast removal of DNA was observed within the first 120 min and remained steady thereafter. The fast removal of DNA by magnetite within the first 120 min shows a very strong interaction between DNA and magnetite surface due to availability of sites for binding onto magnetite. As the contact time increases, bonding sites were observed to be fully occupied thereby causing adsorption rate to be steady. Furtherance, increase in DNA concentration also showed to influence of rate of removal as higher DNA concentration enhances the availability of DNA available for binding onto magnetite.

#### Kinetic study

To understand the mechanism of removal of antibiotics resistant bacterial DNA from wastewater using magnetite, kinetic study was carried out using three kinetic models including pseudo 1st order, pseudo 2nd order and Elovich models (SI Table 1). When the adsorption data obtained from the removal of bacterial DNA from wastewater using magnetite were fitted into the linear form of these three models, the kinetic parameters obtained showed that pseudo 2nd order kinetic model better explain the mechanism of adsorption of DNA from wastewater by using magnetite going by their regression coefficients (*R*^2^) values which are close to 1 (Table 1) and the plot showing the fitting of the adsorption data into the kinetic models is presented in SI Fig. 1. In addition, the *q*_*e*_ calculated for pseudo 2nd order was found to be close to the* q*_*e*_ experimental values for all three DNA concentrations compared with those obtained for the other two kinetic models. The implication of these results is that the removal of bacterial DNA by magnetite was achieved by bimolecular interaction between magnetite and bacterial DNA carrying ARGs. Similar results have been previously reported (Li et al. 2012). When the residual chi-square (*X*^2^) was employed for the determination of the model which better explains the kinetic process, it was found that the pseudo-second-order kinetic model best describes the adsorption of bacterial DNA by magnetite from aqueous solution. Obijole et al. reported that the residual chi square with the lowest amount better explains a process (Obijole et al. 2021). In this study, the *X*^2^ of pseudo-second-order kinetic model has the lowest *X*^2^ compared to those of pseudo-first-order and Elovich kinetic models (Table 1). This result compliments the result obtained for the coefficient of determination in this study.

**Table 1:** Kinetic parameters for the removal of multidrug-resistant bacterial DNA from water

	Pseudo 1st order				Pseudo 2nd order				Elovich				
Initial Conc. (ng/mL)	*q*_*e*_ (calc) ng g^−1^	K_1_ (min^−1^)	*R*^*2*^	*X*^*2*^	*q*_*e*_ (calc) ng g^−1^	K_2_ (min^−1^)	*R*^*2*^	*X*^*2*^	*α* (ng g^−1^ min^−2^)	*β* (g ng^−1^ min^−1^)	*R*^*2*^	*X*^*2*^	*q*_*e*_ exp (ng g^−1^)
**185**	7.55	− 0.0137	0.7384	192.9734	45.59	0.0212	0.9991	0.0002650	41.07	0.102	0.9030	0.5264	**45.72**
**165**	4.38	− 0.0197	0.2076	324.4954	42.06	0.0227	0.9970	0.0000095	37.96	0.560	0.9198	0.4472	**42.08**
**145**	10.22	− 0.0226	0.3934	47.1002	32.19	0.0287	0.9884	0.0000279	18.82	0.0417	0.8336	9.45566	**32.16**

A comparison of the maximum adsorption capacity of magnetite used in this study with other adsorbents already reported in published articles shows that magnetite performed considerably well compared to other adsorbents for the removal of DNA of antibiotic resistant bacterial from water/wastewater (Table 2). This is an indication that magnetite nanoparticles can serve as an effective material for the removal of bacterial DNA from water/wastewater and consequently for the treatment of water conveying antimicrobial resistance determinants.

**Table 2:** Comparison of the adsorption capacity, *q*_*e*_for multidrug-resistant bacterial DNA removal by magnetite nanoparticles and other materials in literature

Adsorbents	Conditions	*q*_*e*_	References
CeO nanoparticle	pH 5.50, 10.10 µg/mL DNA conc, 25 °C, 300 min	1.947 µg g^−1^	Anthony et al. (2020)
Silver nanoparticles	pH 6.0, 25 mL of 14.98 µg/mL DNA solution, room temp., 20 mg adsorbent	4.21 µg g^−1^	Ezeuko et al. (2022b)
Iron oxide nanoparticles	pH 7.6, 50 nM adsorbate, 298 K, 2.5 µg adsorbent 24 h	30 nM	Liu and Liu (2014)
Mesoporous silica	pH 6.2, 20 mL of 10 µg/mL DNA solution, room temp., 10 mg adsorbent	3.7 µg g^−1^	Li et al. (2012)
Magnetite nanoparticles	pH 6.0, 185 ng/mL, 10 mL DNA solution, 22 °C, 20 mg adsorbent, 6 h	45.5 ng g^−1^	Current study

#### Effect of adsorbent weight

**Fig. 6:**
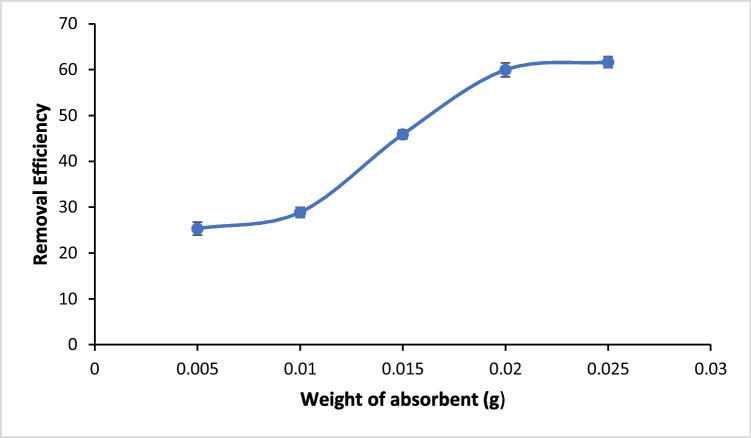
Effect of adsorbent weight on the removal of multidrug-resistant bacterial DNA from water (Conditions: pH 6, DNA concentration of 165 ng/mL, 20 °C, 200 mM NaCl, and 6 h contact time)

The effect of adsorbent weight on the removal of bacterial DNA harboring ARGs from wastewater by magnetite was carried out using five different magnetite weight, 0.005, 0.01, 0.015, 0.02, and 0.025 g at fixed DNA concentration of 185 ng/mL and pH 6. The adsorption study showed that effect of adsorbent weight alters the rate of adsorption of bacterial DNA (Fig. 6). It can be seen that DNA removal increases (~ 60% removal efficiency) as magnetite dose increases to 0.02 g and remained stable to 0.025 g. This result is connected to the fact that as magnetite weight increases, the number of active sites on the surface of magnetite available for the removal of antibiotic-resistant bacterial DNA also increases resulting into an increase in percentage removal of DNA thereby resulting into occupation of magnetite’s surface and decreasing its total surface area. However, between 0.02 and 0.025 g, the removal efficiency remained stable; this can be explained by the fact that lesser amounts of DNA are left in solution for adsorption onto magnetite thereby impeding higher removal of efficiency

#### Effect of cations on the removal of bacterial DNA harboring ARGs from water

**Fig. 7:**
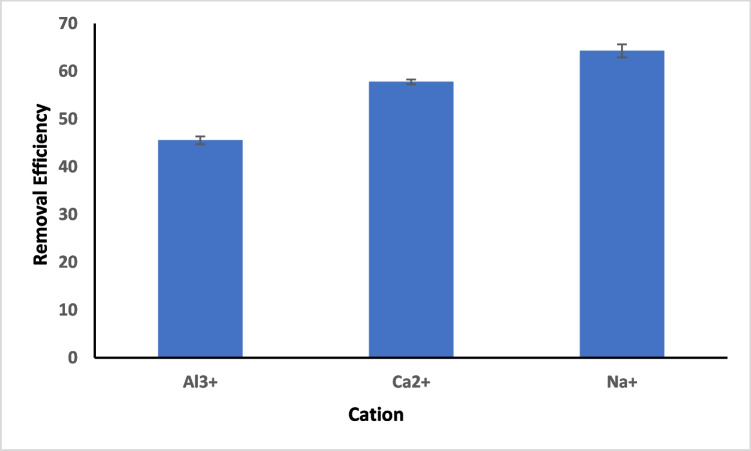
Effect of cation on the removal of multidrug-resistant bacterial DNA from aqueous solution(Conditions: 165 ng/mL DNA solution,20 °C, 200 mM NaCl, 0.02 g magnetite dosage, pH 6, and 6 h contact time)

The effect of cations on the removal of bacterial DNA harboring ARGs from water was carried out in this study. The reason for this experiment was to ascertain the effectiveness of magnetite to remove bacterial DNA in the presence of positive ions in solution. Three cations namely Na^+^, Ca^2+^, and Al^3+^ were used to contaminate water containing a known concentration of DNA. The result obtained (Fig. 7) indicates that the presence of cations in solution positively influences the adsorption efficiency of magnetite. Solutions containing metal ions with lower oxidation states were observed to show higher removal of DNA from solution by magnetite. The reason for the observation is due to the complexation of DNA by these cations to the negative ion backbone in the DNA structure. Similar result has been previously published by Radi et al. (2016). Consequently, cationic ions with lower valence electrons readily bind to the surface of DNA than higher valence electrons cations; this might be ascribed to their higher position in the electrochemical series and low ionization energies. This study shows a percentage efficiency of 64.31, 57.78, and 41.55% for DNA removal by magnetite in the presence of Na^+^, Ca^2+^, and Al^3+^ respectively.

#### Removal of multidrug-resistant bacterial DNA from real wastewater

To evaluate the practical efficacy of magnetite nanoparticles in treating actual wastewater, we conducted adsorption studies on water samples sourced from distinct municipal wastewater treatment plant in Alice. These samples, characterized by an average antibiotic-resistant bacterial DNA concentration of 14 ng mL^*−*1^, were spiked with DNA solution to attain 85 ng mL^*−*1^ DNA concentration in the wastewater. Under optimal conditions (pH 6.0, 200 mM NaOH solution, adsorbent dose 20 mg, 120 min, and 20 mL of DNA solution), the results revealed an average adsorption efficiency of 53% for the real wastewater samples.

The observed lower adsorption efficiency in real wastewater samples may be attributed to the presence of competing ions and organic contaminants vying for binding with the active sites of magnetite alongside DNA. This competition likely hampers the adsorption process in real-world scenarios. (Anthony et al. 2022) noted a similar phenomenon, indicating that the presence of competing ions in real wastewater samples leads to a reduction in the adsorption efficiency of the target contaminant. This finding aligns with our own observations.

In light of these results, we conclude that while the adsorption efficiency of magnetite for real wastewater is slightly below that of simulated water, it still exhibits a notable adsorption capability. This capacity allows for the selective removal of DNA from domestic and industrial wastewater, even in the presence of various contaminants. Thus, our findings affirm the potential of magnetite as a promising adsorbent for the effective removal of multidrug-resistant bacterial DNA from diverse wastewater sources.

## Conclusion

This study evaluates the efficacy of magnetite to remove multidrug resistant bacterial DNA from wastewater with the aid of NaCl salt. Prior to adsorption experiments, synthesis and characterization of the nanomaterial were carried out and characterization result showed that magnetite was successfully synthesized by precipitation method. Adsorption studies showed that pH, time, and DNA initial concentration play a vital role in the removal of bacterial DNA from wastewater. Kinetic study showed that the removal of bacterial DNA conveying ARGs from wastewater by magnetite followed pseudo-second-order model and was at the phosphate end of DNA. In addition, this material and adsorption process effectively removed DNA from real municipal wastewater samples with removal efficiency of 53%, an indication of the potential of magnetite to be applicable for the removal of multidrug-resistant bacterial DNA and by implication antibiotic resistance genes from water/wastewater. Finally, considering the influence of other operating conditions such as COD, BOD, chlorine availability, water flow, removal mechanisms, genetic conjugation, transformation and transduction, mutation, and selection is crucial when employing this material in the treatment of wastewater harboring ARGs in real sewage plants.

## Supplementary Information

Below is the link to the electronic supplementary material.

ESM 1 (PPTX 130KB)

## Data Availability

Data generated in this study will be made available on request.
