# Supplementary material for: Effectiveness of magnetite nanoparticles for the removal of DNA of multidrug-resistant Escherichia coli from municipal wastewater
Source: Environ Sci Pollut Res Int. 2024 Sep 30;32(54):30135–45. doi: 10.1007/s11356-024-35098-5 (PMC12804236; doi:10.1007/s11356-024-35098-5)
Supplement: Supplementary file 1 — (PPTX 130KB) [file 11356_2024_35098_MOESM1_ESM.pptx]

## Slide 1
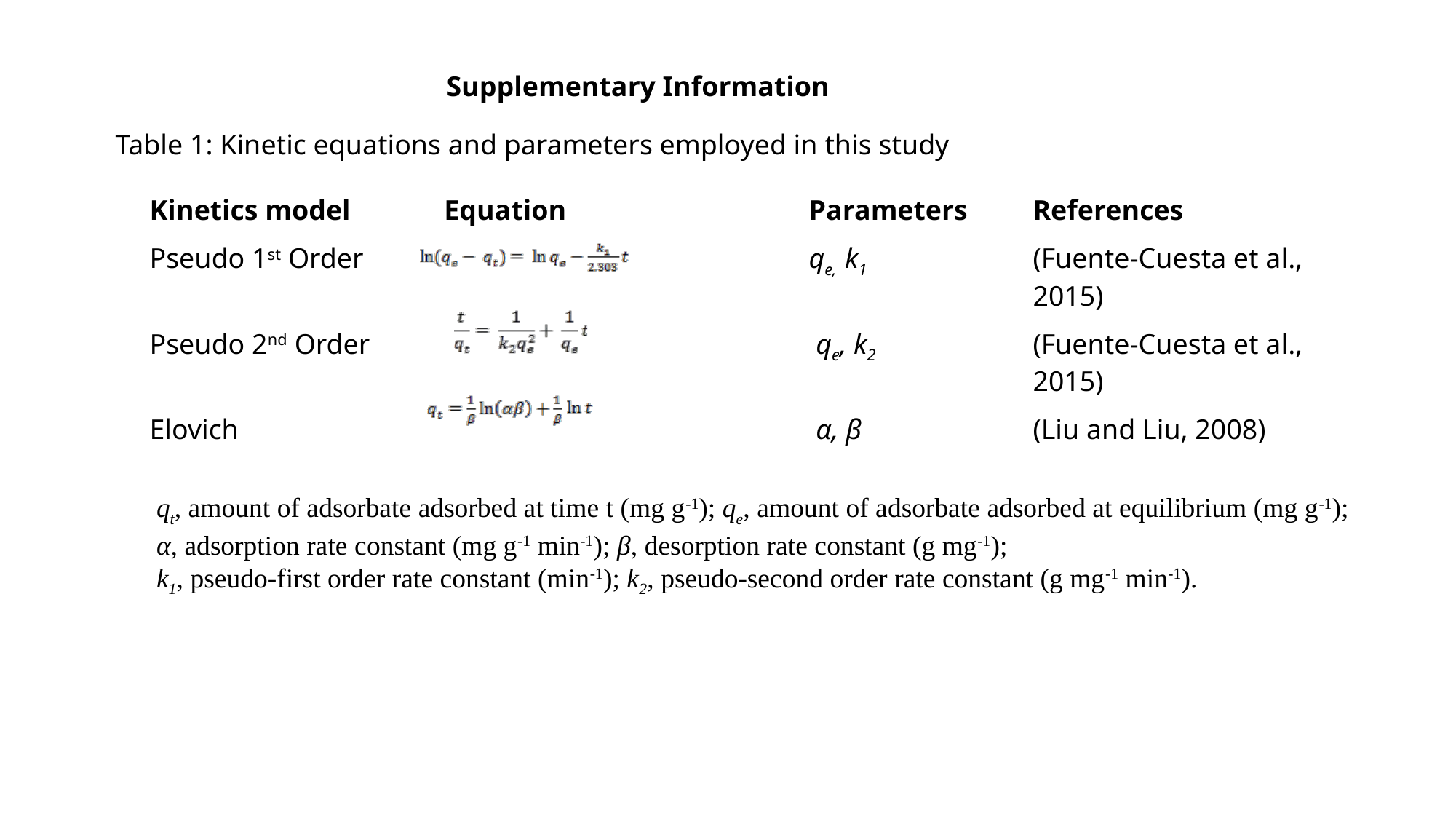

Supplementary Information
Table 1: Kinetic equations and parameters employed in this study
| Kinetics model | Equation | Parameters | References |
| --- | --- | --- | --- |
| Pseudo 1st Order | | qe, k1 | (Fuente-Cuesta et al., 2015) |
| Pseudo 2nd Order | | qe, k2 | (Fuente-Cuesta et al., 2015) |
| Elovich | | α, β | (Liu and Liu, 2008) |
qt, amount of adsorbate adsorbed at time t (mg g-1); qe, amount of adsorbate adsorbed at equilibrium (mg g-1);
α, adsorption rate constant (mg g-1 min-1); β, desorption rate constant (g mg-1);
k1, pseudo-first order rate constant (min-1); k2, pseudo-second order rate constant (g mg-1 min-1).

## Slide 2
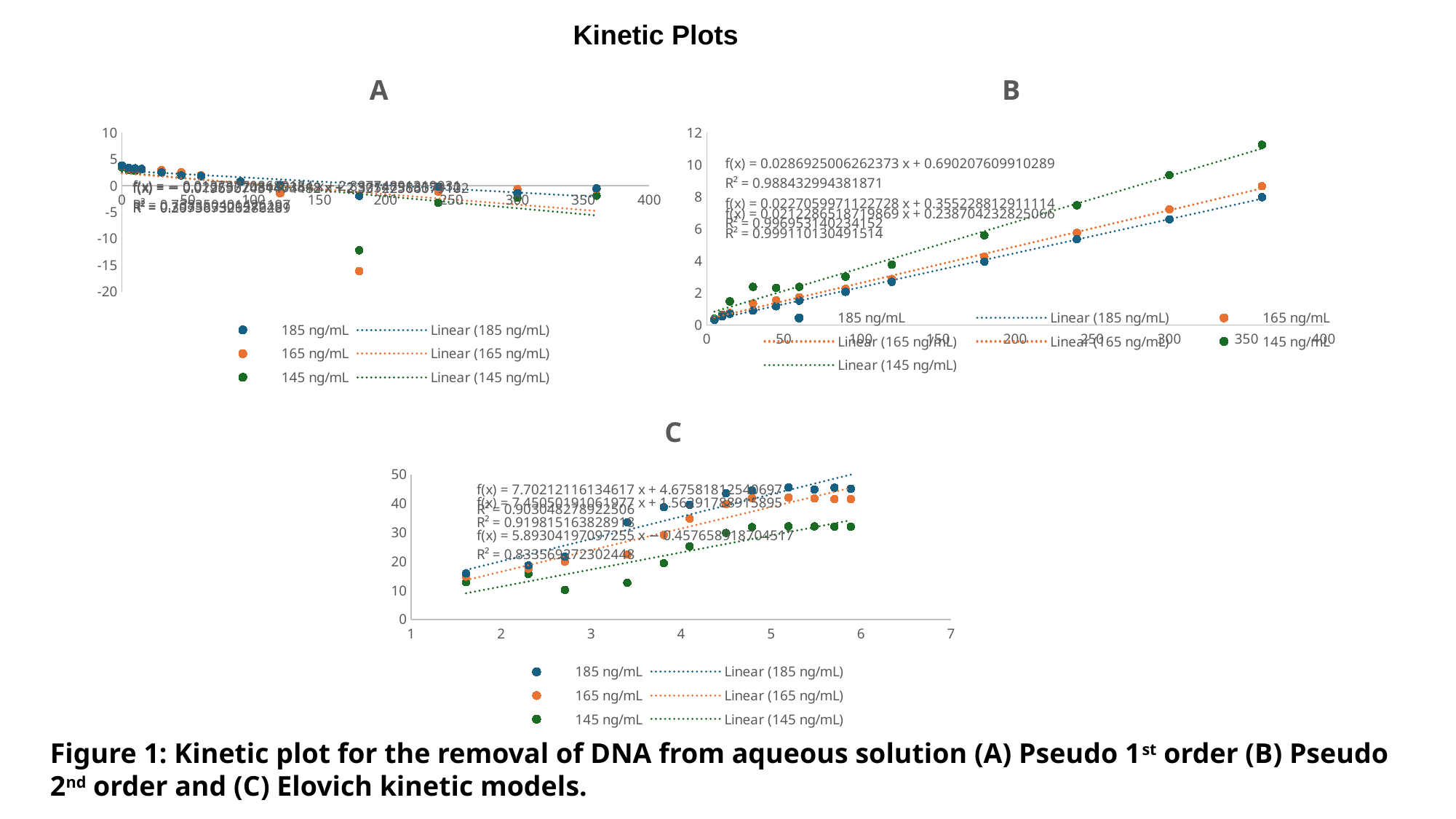

Kinetic Plots
### Chart: B
| Category | 185 ng/mL | 165 ng/mL | 145 ng/mL |
|---|---|---|---|
### Chart: A
| Category | 185 ng/mL | 165 ng/mL | 145 ng/mL |
|---|---|---|---|
### Chart: C
| Category | 185 ng/mL | 165 ng/mL | 145 ng/mL |
|---|---|---|---|Figure 1: Kinetic plot for the removal of DNA from aqueous solution (A) Pseudo 1st order (B) Pseudo 2nd order and (C) Elovich kinetic models.

## Slide 3
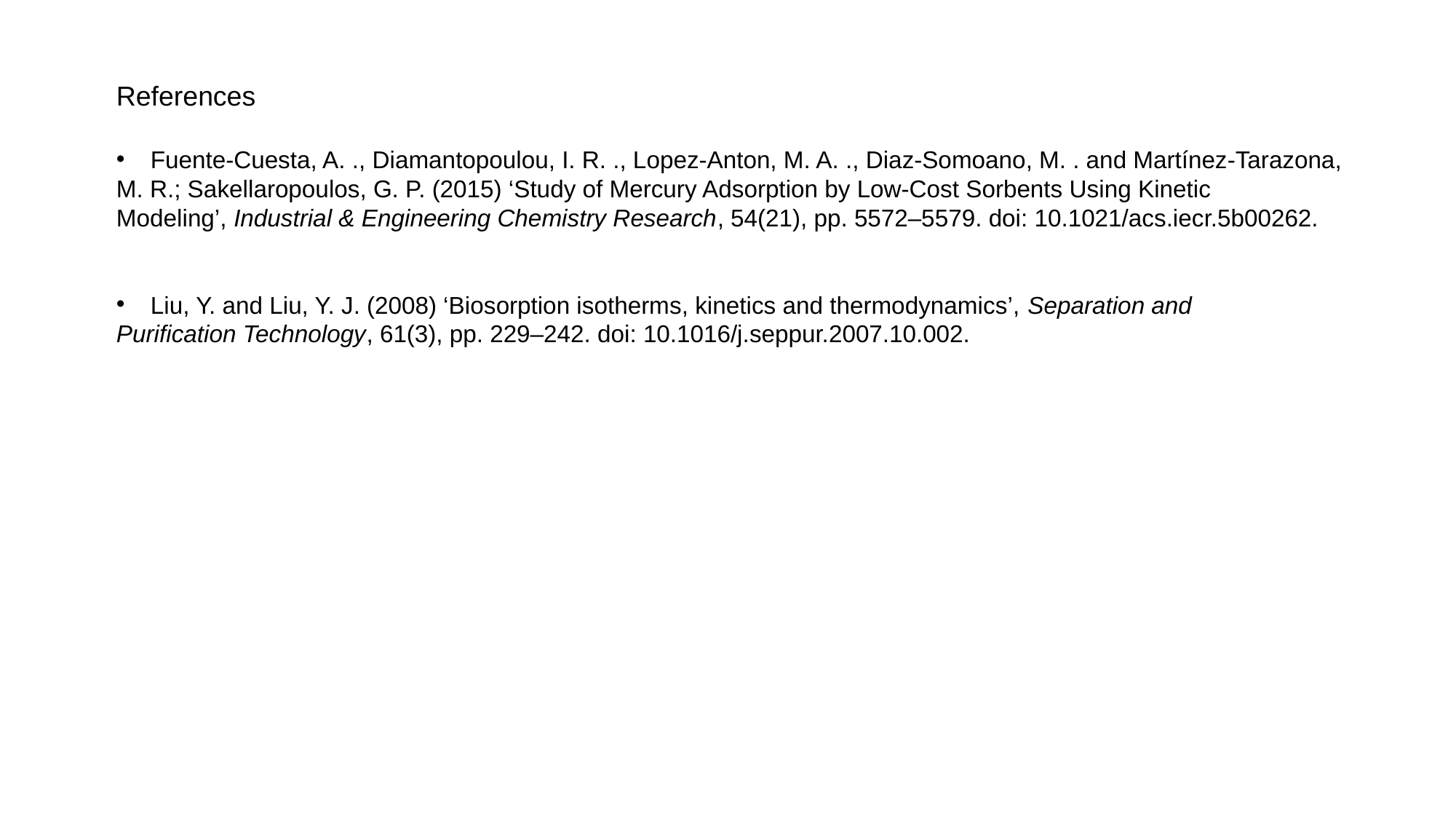

References
Fuente-Cuesta, A. ., Diamantopoulou, I. R. ., Lopez-Anton, M. A. ., Diaz-Somoano, M. . and Martínez-Tarazona,
M. R.; Sakellaropoulos, G. P. (2015) ‘Study of Mercury Adsorption by Low-Cost Sorbents Using Kinetic
Modeling’, Industrial & Engineering Chemistry Research, 54(21), pp. 5572–5579. doi: 10.1021/acs.iecr.5b00262.
Liu, Y. and Liu, Y. J. (2008) ‘Biosorption isotherms, kinetics and thermodynamics’, Separation and
Purification Technology, 61(3), pp. 229–242. doi: 10.1016/j.seppur.2007.10.002.
